# Fabrication of Porous Spherical Beads from Corn Starch by Using a 3D Food Printing System

**DOI:** 10.3390/foods11070913

**Published:** 2022-03-22

**Authors:** Safoura Ahmadzadeh, Ali Ubeyitogullari

**Affiliations:** 1 Department of Food Science, University of Arkansas, Fayetteville, AR 72704, USA; safouraa@uark.edu; 2 Department of Biological and Agricultural Engineering, University of Arkansas, Fayetteville, AR 72701, USA

**Keywords:** 3D food printing, starch, beads, rheology, porous structure, textural properties

## Abstract

This study introduces a 3D food printing approach to fabricate spherical starch beads with small sizes and high porosity for the first time. The results illustrated that 3D food printing could generate starch beads in different sizes depending on the nozzle diameter, printing pressure, and ink viscosity. The 3D-printed beads were characterized for their morphology, crystallinity, and textural properties, while the starch-based ink was analyzed for its rheological properties. A suitable printing was attained when viscosity was in the range of 1000–1200 Pa.s at a low shear rate (˂0.1 s^−1^). Among the starch concentrations (10–15%, *w*/*w*) investigated, 15% starch concentration provided the best control over the shape of the beads due to its high storage modulus (8947 Pa), indicating higher gel strength. At this condition, the starch beads revealed an average size of ~650 µm, which was significantly smaller than the beads produced with other starch concentrations (10 and 12.5%), and had a density of 0.23 g/cm^3^. However, at lower starch concentrations (10%), the beads were not able to retain their spherical shape, resulting in larger beads (812–3501 µm). Starch crystallinity decreased by gelatinization, and the starch beads exhibited a porous structure, as observed from their SEM images. Overall, 3D food printing can be an alternative approach to preparing porous beads for the delivery of bioactive compounds with high precision.

## 1. Introduction

Advanced materials with unique properties such as nanoporous structures have received great attention due to their potential applications in modern high-tech fields, including biotechnology and food industries [1]. Potential advanced applications of porous materials in the food industry consist of the delivery of bioactive compounds for enhanced bioavailability and intelligent packaging for extending shelf-life [2]. In delivery systems, carriers/encapsulants are generally designed to increase the bioavailability of sensitive bioactive compounds by protecting them from degradation during processing, storage, and digestion [3]. The performance of the carriers strongly depends on their size, shape, surface chemistry, and stability. Moreover, for food applications, using only food-grade materials as carriers is vital for the successful adoption of the delivery method.

Among food-grade biopolymers, starch has received great attention due to its ability to create a 3D open porous structure by simply gelatinization upon heating. In addition, starch can be easily incorporated into various food formulations since it is abundant, non-toxic, inexpensive, biocompatible, and part of many food systems [4,5]. Starch composes of amylose, a linear polymer of α(1→4) linked D-glucose units, and amylopectin, a branched polymer with α(1→4) and α(1→6) bonds, which readily forms a gel network through a three-step process consisting of swelling, gelatinization, and retrogradation. Compared to native starches, high amylose corn starches provide health benefits due to them having a higher amount of resistant starch, and they are gluten-free [6]. Moreover, encapsulants developed based on high amylose starches have more efficient matrices for the delivery of bioactive compounds as they have higher porosities [7]. Therefore, in this study, high amylose corn starch was used to create porous beads.

Starch hydrogels were successfully shown to increase the bioaccessibility of bioactive compounds such as curcumin [2] and polyphenols [8]. Starch hydrogel microspheres for bioactive compound encapsulation were conventionally produced using emulsion formulations [8,9]. However, this emulsification approach utilizes surfactants and a high amount of oil, which are later required to be removed using a solvent extraction method. The use of organic solvents and surfactants and the need for the additional separation step require high energy limit food and pharmaceutical applications of this conventional approach. Therefore, there is a need for a novel method to generate starch hydrogel beads before realizing their full potential in bioactive compound delivery. 

Additive manufacturing (AM) or 3D printing has recently been adapted to manufacture food products [10]. AM technology was originally devised to fabricate 3D complex objects in a single step. This technology has the potential to fabricate specific foods to meet individual needs by regulating their nutrient content and composition [11]. 

Thus far, 3D food printing has been employed to create 3D geometric shapes of basic food formulations such as potato puree, chocolate, meat paste, cheese, and dough [12,13]. Compared to conventional food production methods, 3D food printing as a bottom-up technique is more flexible, less expensive, and minimizes waste production. Moreover, unique properties such as the highly complex geometry of products that were previously inaccessible can be obtained by using 3D food printing technology [1].

In addition to creating macroscopic geometric shapes, 3D food printing can be used to precisely control the microstructure of foods. When the technologies of 3D food printing and functional porous materials (i.e., starch hydrogels) are combined, their performance, structure, and application scope can be broadened, especially where intricate structures and shapes are desired. Direct printing of an object with a porous structure is critically challenging, which was recently addressed using polymers with inherent porosity [1,14,15]. The porous interior structure, including pore size, needs to meet the requirements for a particular application, which can be controlled in printed objects via modifying processing parameters, including the concentration and composition of the ink.

The sol–gel method, the main procedure typically applied to make polymer-based hydrogels, can be simply adapted to formulate ink suitable for 3D printing technologies. However, given the demand for both biological function and physical characteristics, the utilization of biopolymers for 3D printing is highly challenging [5,14,15]. Recent studies illustrated the adaptability of starch-based hydrogels for 3D food printing technology [16], where 15–50 mm starch hydrogels were 3D-printed for promoting cell growth. However, there is no existing study reporting the formation of starch hydrogel beads using 3D food printing technology. Therefore, the objective of this study was to develop and optimize a 3D food printing approach to fabricate starch beads. In this research, a protocol for generating starch hydrogel beads by extrusion-based 3D food printing was developed and optimized for the first time. The fabrication process of the porous starch beads via 3D printing involved three steps: (i) forming a highly consistent starch paste with appropriate rheological properties, (ii) extrusion-based 3D food printing, and (iii) freeze-drying. In this study, the 3D printing parameters (i.e., nozzle diameter, printing pressure, and starch concentration) were optimized for the smallest bead size of the 3D-printed starch hydrogel beads. The effects of nozzle diameter and starch concentration on bead size morphological, structural, and thermal properties of starch beads were investigated. Structural integrity, printing accuracy, and consistency were also considered in determining the optimum printing parameters.

## 2. Materials and Methods

### 2.1. Materials

High amylose corn starch (HACS) with 72% amylose content and 11% moisture content (Hylon VII) was kindly obtained from Ingredion (Westchester, IL, USA) and used as the raw material to generate beads.

### 2.2. Preparation of Starch Beads by Extrusion-Based 3D Food Printing

First, aqueous starch suspensions of three concentrations (10, 12.5, and 15%, *w*/*w*) were prepared. These starch concentrations were selected based on the preliminary 3D food printing experiments. The samples were then heated up to 95 °C under high shear (4260 rpm) using a Thermomix (Vorwerk, Thousand Oaks, CA, USA) and maintained at this temperature for 25 min, and then immediately used as an ink for 3D food printing.

**Figure 1:**
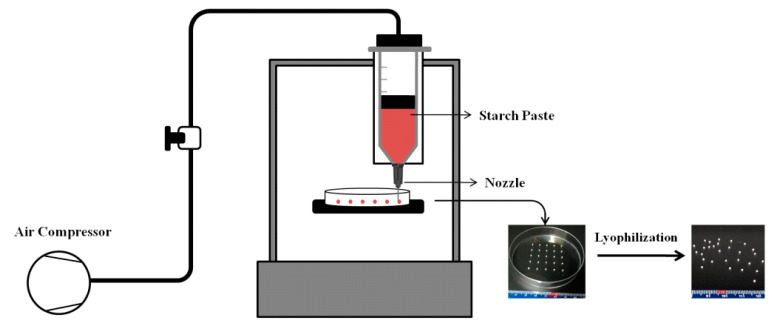
Schematic diagram of the extrusion-based 3D food printing system.

Three-dimensional food printing was carried out using an Allevi 2 Bioprinter (Allevi, Inc., Philadelphia, PA, USA) that was equipped with two 10 mL extruders. The ink extrusion was empowered by pneumatic pressure (Figure 1).

Next, the developed starch paste was loaded into a 10 mL cartridge, which was then transferred to one of the extruders of the 3D printer previously warmed up to 95 °C. The starch hydrogel was then extruded through nozzles with different diameters (i.e.,23, 25, 30, 32, and 34 G corresponding to 0.330, 0.250, 0.152, 0.100, and 0.080 mm internal diameters, respectively) (Table 1). The nozzle height from the printing plate was adjusted as the double nozzle diameter.

During printing, the cartridge temperature was kept constant at 95 °C. The extrusion pressure was optimized for each printing condition (i.e., starch concentration and nozzle diameter) in the range between 0.027 and 0.17 MPa. The printing speed was kept constant at 6 mm/s, and the Bioprint Pro software was used to print spherical beads. The starch-based ink was deposited at room temperature (23 °C). Retrogradation was performed by keeping the gel beads at 4 °C, and then the beads were transferred to a freezer −80 °C, and finally dried for 24 h at −108 °C using a freeze dryer (VirTis benchtop SLC, Warminster, PA, USA). High amylose corn starch and the 3D-printed starch beads with starch concentrations of 10, 12.5, and 15% were thereafter referred to as HACS, SP-10, SP-12.5, and SP-15, respectively. All 3D food printing experiments were conducted in triplicates.

### 2.3. Macroscopic and Microstructural Observations

The bead size and sphericity of the 3D-printed starch beads were determined from their images using ImageJ software (public domain, National Institutes of Health, Bethesda, MD, USA). Randomly selected 50 beads were used to determine the mean bead size ± standard deviation and sphericity ± standard deviation.

FEI NovaNanolab200 (FEI company, Hillsboro, OR, USA) Dual-Beam system equipped with a 30 kV SEM FEG column and a 30 kV FIB column was utilized to visualize the structure of the starch beads. The specimens were prepared by cutting cross-sections from the starch beads, and then they were sputter-coated with a gold layer (EMITECH SC7620 Sputter Coater, Fall River, MA, USA) to prevent electrical charging. Finally, the SEM images were taken at an acceleration voltage of 10 kV and a current of 10 mA.

Furthermore, the macropore size distribution was determined from the SEM images by measuring the size of randomly selected 50 open pores using ImageJ software. The thickness of the beads’ surface was also measured using ImageJ software.

### 2.4. Density and Porosity

The weight of a certain amount of the starch beads was divided by their volume to calculate the bulk density (ρ_b_). The volume of the beads was determined via measuring the beads’ dimensions using ImageJ software (public domain, National Institutes of Health, Bethesda, MD, USA). The true density was measured using a helium pycnometer (Accupyc1340, Norcross, GA, USA). Finally, the porosity (ε) was then determined using the bulk and true density [17].

### 2.5. Fourier Transform Infrared Spectroscopy (FTIR)

The structural features of HACS and starch beads were investigated using an IRAffinity-1S Fourier transform infrared spectroscopy (FTIR) unit (SHIMADZU Corp., Kyoto, Japan) equipped with a Quest attenuated total reflectance (ATR) accessory (Specac Company, Orpington, UK). The FTIR spectrum was acquired in the range of 4000 to 400 cm^−1^ at a resolution of 4 cm^−1^ with 64 scans [18].

### 2.6. X-ray Diffraction

The crystalline structure of the 3D-printed starch beads was evaluated through an X-ray diffraction (XRD) analysis. The XRD patterns were recorded using a PW3040X’PertMRD

High-Resolution X-ray diffractometer (Philips, Almelo, Netherlands). The powdered starch beads were packed into the sample holder, and then they were scanned from 5° to 40° (2*θ*) with a step size of 0.02 at 45 kV and 40 mA [19]. The XRD patterns were then used to calculate the degree of crystallinity following the method of Ubeyitogullari and Ciftci [19]. The relative crystallinity was calculated by dividing the area under the crystalline peaks to the total area under the curve (i.e., both the crystalline peaks and amorphous region).

### 2.7. Thermal Properties

Differential scanning calorimetry (DSC) measurements were performed using a PerkinElmer DSC 4000 calorimeter (Waltham, MA, USA). Briefly, 8 μL of deionized water was added to 3.5 mg (dry basis) of powdered starch beads and sealed in a 50 μL aluminum pan. The DSC measurements were then conducted in a heating cycle from 25 °C to 150 °C at a rate of 5 °C/min under a nitrogen atmosphere. The DSC data were used to determine the gelatinization temperature as well as the melting point of amylose–lipid complexes.

### 2.8. Rheological Properties

A controlled-stress rheometer (AR 2000 Rheometer, TA Instruments, New Castle, DE, USA) equipped with a Peltier Plate temperature-controlled system and a 40 mm parallel-plate geometry was used to measure the rheological properties. Rheological measurements were performed according to the method reported by Chen et al. [16] with minor modifications. Apparent viscosity was recorded by undertaking steady shear flow tests in the shear rate range of 0.1–100 s^−1^ at 95 °C.

The viscoelastic properties of starch hydrogels were determined by using storage modulus (G′), loss modulus (G″), and loss tangent (tan δ, G″/G′) as the dynamic mechanical parameters. Temperature sweeps were performed by preparing a starch suspension with a certain concentration (10, 12.5, or 15%, *w*/*w*) and loading it between the parallel plate geometry with a 40 mm diameter and 1 mm gap. The moisture evaporation from the samples was prevented by covering the edge of the samples with silicon oil. Afterward, the temperature sweeps were carried out from 50 °C to 95 °C at a rate of 2°/min with 0.01% strain and 10 rad/s frequency.

For the oscillation tests, starch hydrogels were prepared by heating the starch suspension with a certain concentration up to 95 °C and keeping it at that temperature for 10 min. The linear viscoelastic region (LVR) was first determined by conducting strain sweeps at a frequency of 10 rad/s. The oscillatory stress sweeps were then performed at the same frequency (10 rad/s) in order to measure yield stress (τy) and flow stress (τf). The stress value related to a 10% drop in G′ value was reported as the yield stress (Pa). Moreover, the crossover point of G′ and G″ was considered as the flow stress. The angular frequency sweep test was carried out by increasing the frequency from 0.1 to 100 rad/sat 95 °C. The average of three replications was reported for each rheological parameter.

### 2.9. Statistical Analysis

The analysis of variance (ANOVA), followed by the LSD comparisons of means test (*p* ≤ 0.05), was performed using SPSS Statistics software. The average of three replications along with their standard deviations was reported for the measured parameters.

## 3. Results and Discussion

### 3.1. Macroscopic and Microstructural Observations

**Figure 2:**
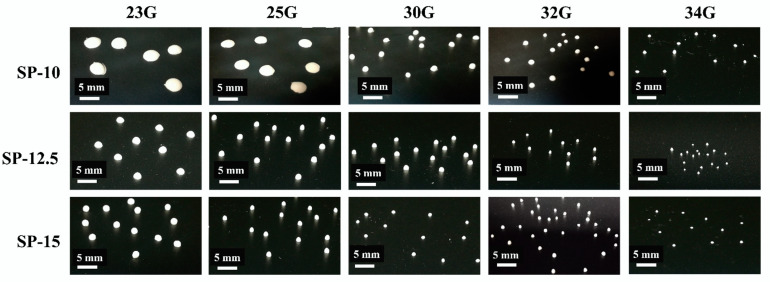
Pictures of the 3D-printed beads produced using starch concentrations of 10, 12.5, and 15% (*w*/*w*) and nozzles with different diameters (23, 25, 30, 32, and 34 G).

In creating 3D-printed beads, the ability of the ink to hold the desired shape and size after printing is as important as the technical capabilities during printing, such as the printing resolution of the 3D food printer. Therefore, the printing parameters, including starch concentration, and nozzle size, were investigated, and the mean bead sizes and sphericities of the 3D-printed beads were determined (Table 2). The images of the 3D-printed starch beads are shown in Figure 2, illustrating the excellent 3D printing consistency. 

The bead size of starch beads significantly (*p* < 0.05) decreased with decreasing the nozzle diameter and increasing the starch concentration. Regarding the size and sphericity of the starch beads, the best results were obtained with a 15% starch concentration and nozzle 34G (Figure 2). As depicted in Table 2, the sphericity for SP-15 is close to 1, indicating that the beads are almost spheres. Nevertheless, the sphericity of SP-12.5 and especially SP-10 significantly deviated from 1. The results confirmed that by increasing the starch concentration, the resulting bio-ink enabled better retention of the spherical shape. However, the 3D printability could be maintained as long as the starch concentration, as the dominant factor, was higher than 10%. The preliminary experiments indicated that starch concentrations higher than 15% resulted in very high mechanical strength, preventing the formation of spherical beads, where the starch paste formed strip shape while printing and was not able to form spherical beads (data not shown). García-González et al. [9] reported that via the emulsion–gelation method, the sphericity of the particles was improved by storing the samples at low temperature, resulting in better retrogradation. They also reported that the higher ratio of oil to starch solution and the presence of surfactant improved the sphericity of the particles. In another study, Wang et al. [8] reported that via the dispersion–inverse gelation process, the distance between the needle and the oil phase affected the sphericity of the starch particles. They reported that by increasing this distance to 10 cm, more spherical particles were obtained.

The average diameter of starch beads significantly decreased from 812 to 650 μm by increasing the starch concentration from 10 to 15% at a similar set of printing parameters (*p* < 0.05). At the same starch concentration, nozzle size and printing pressure played important roles in achieving the desired bead size. Smaller nozzle diameters resulted in smaller bead sizes; however, higher printing pressure was required to create beads (Figure 2). Previously, Wang et al. [8] generated starch beads using a dispersion–inverse gelation process where the starch paste was formed by heating a starch suspension at 100 °C for 30 min, and then it was added dropwise to soybean oil to create starch hydrogel beads. The size of the starch hydrogel beads ranged between 1.71 and 1.83 mm, which is ~3 fold larger than the smallest beads obtained in this study. However, several ethanol washes were required to remove the soybean oil from the starch beads. In another study, García-González et al. [9] obtained smaller beads (215–1226 μm range), using an emulsion–gelation method in which oil:starch emulsions were formed and heated up to the high temperatures (95–140 °C) in a closed system (~0.2 MPa) while stirring. Finally, after depressurization and cooling, the oil phase was removed by centrifugation and several ethanol washes. The authors reported that the high shear (i.e., 1400 rpm) upon cooling has drastically affected the bead size; a higher stirring rate has created smaller beads.

In this study, depositing hot starch paste (95 °C) at room temperature allowed self-supporting of the starch paste and, in turn, retained the spherical shape without further curing process [21]. Therefore, monodisperse microspheres can be synthesized via optimizing starch concentration, 3D printing pressure, and nozzle size. Nevertheless, the starch gel strength played the primary role in spherical bead formation: the stronger the hydrogel, the better retention of its 3D shape. Precise results were obtained by keeping the pressure stable while printing. Since the starch had already been gelatinized, the ink viscosity was expected to be stable during the printing, as confirmed by printing monodisperse beads. When using one specific nozzle diameter, printing pressure and ink viscosity were positively correlated, as the ink with higher viscosity needed higher pressure to be printed; as expected, when using a smaller nozzle diameter, higher pressure was required to print the starch paste with a certain starch concentration.

**Figure 3:**
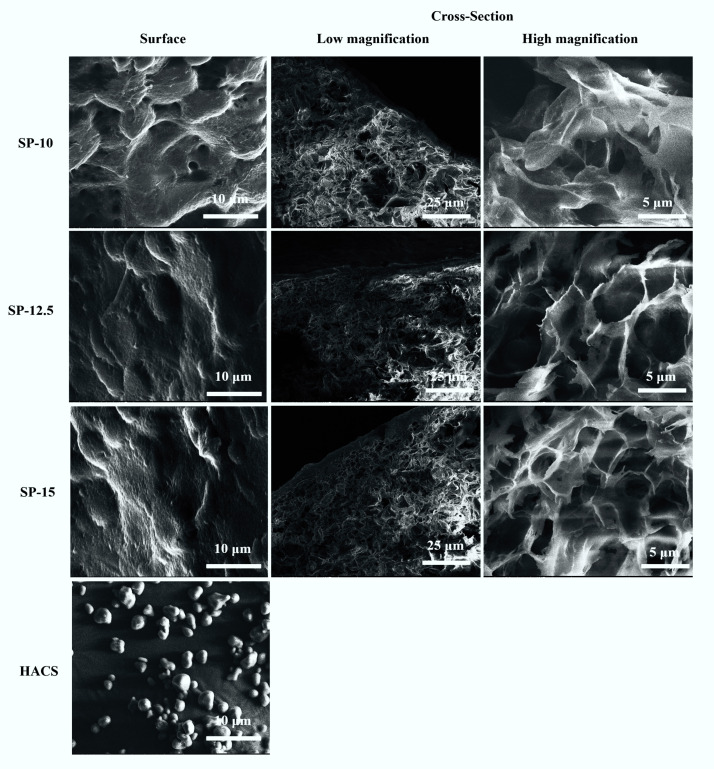
FE-SEM images taken from the surface and cross-section of the 3D-printed starch beads generated using starch concentrations of 10, 12.5, and 15% (*w*/*w*).

The morphology of the 3D-printed starch beads is depicted in Figure 3. As shown in the SEM images, starch beads prepared using HACS consisted of a porous matrix in which nonporous granule residues do not exist.

The granule remnants can be a particular concern when developing small porous spheres. It is important to fabricate homogeneous structures without any granule residues for future applications such as bioactive compound delivery. The heating of starch granules dispersed in an excess amount of water causes irreversible swelling of the granules and amylose dissolution. Complete dissolution of granules occurs only if high enough temperature and shear are used [22]. As reported by Putseys et al. [23], granule residues in corn starch suspensions are not dissolved by only increasing the heating time, indicating the persistence of the granules during heating. In this study, HACS was processed at 95 °C using a high-shear mixer, which improved solubilization of granules and production of a homogeneous matrix pertinently applied to generate porous spheres.

**Figure 4:**
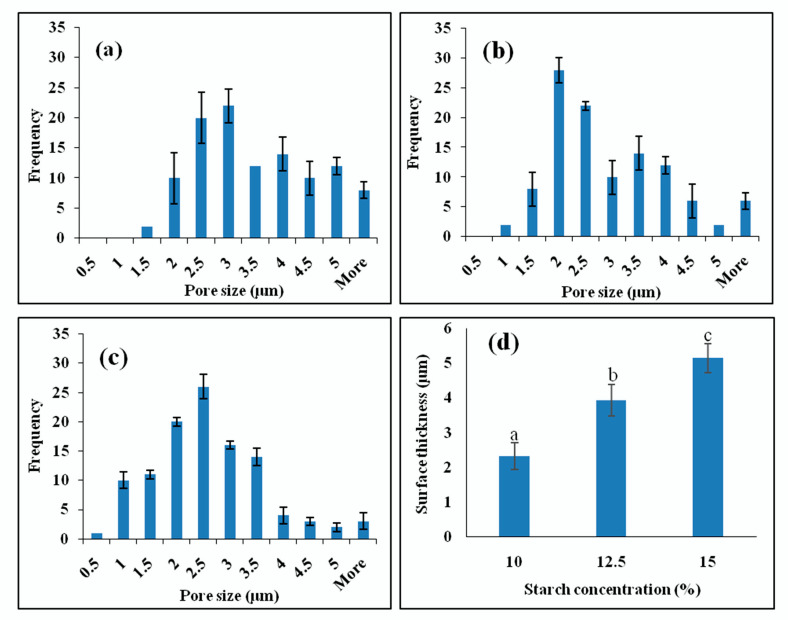
Pore size distribution of the 3D-printed starch beads produced using starch concentrations of (**a**) 10, (**b**) 12.5, and (**c**) 15% (*w*/*w*), and (**d**) their surface thickness (N = 3).

The surfaces of the 3D-printed starch beads are also shown in Figure 3. A dense skin without any pores was formed at the free surface due to the collapsing of the starch matrix at the starch-air interface because of the water evaporation resulting in interfacial tension that generates surface skin [7,24]. The thickness of this layer covering the surface of the beads significantly increased from 2.3 to 5.1 µm (Figure 4d) as the starch concentration increased from 10 to 15% (*w*/*w*) (p < 0.05). This may provide advantages in protecting and delivering sensitive bioactive compounds. The dense surface of the beads may function as a coating layer leading to a controlled release as it covers the internal open-pore structure. Moreover, the release of the loaded compounds can vary based on the thickness of the surface. Previously, Schroeter et al. [25] reported that coating cellulose aerogels resulted in a more controlled drug release.

The starch beads prepared in this study showed porous structure similar to the results reported by Zou and Budtova [7], who prepared porous starch materials via retrogradation of starch solution followed by supercritical CO_2_ drying or lyophilization. They concluded that the main parameter greatly affecting the aerogel properties was the amylose/amylopectin ratio. Furthermore, it was reported that during retrogradation, semi-crystalline clusters are formed through the association of amylose into parallel double helices linked via amorphous regions [26]. At the primary stage of retrogradation, semi-crystalline clusters form a weak gel network with large pores. Subsequently, the crystallinity and gel strength increase due to the formation of more junction zones by amylose chains. This phenomenon can be amplified by higher amylose content [7,24], which was one of the reasons for using HACS in this study, in addition to its rheological properties and resistant starch component.

In this study, freeze-dried starch beads indicated an intricate network with a porous structure (Figure 3). This structure revealed the formation of ice crystals and their growth during freezing, which compressed the starch matrix and resulted in the formation of macropores [22]. For all three concentrations, the internal structure throughout the bead was porous. However, starch concentration affected the size and distribution of pores in the 3D-printed beads. As seen from the SEM images (Figure 3), higher starch concentration produced a more compact network. Similar findings were previously reported for starch and cellulose aerogels [27,28].

Furthermore, the macropore size distribution, ranging between 0.5 and 5 µm, is presented in Figure 4. The pore sizes reported for starch cryogels in the literature [29,30] are different from those observed in this study, which can be related to the preparation method and the intrinsic properties of HACS that influence the morphology. High starch concentration reduced the matrix pore size, which can be due to the gel formation that occurs more rapidly at higher concentrations resulting in denser starch networks. Moreover, at higher starch concentrations, the starch hydrogels are stronger and have less water entrapped in their structure, increasing their stability against ice crystals’ growth.

As mentioned above, the macroporous structure observed in this study was created through freeze-drying, which can be improved by using supercritical CO_2_ drying, where beads with finer networks can be generated, granting them more potential in being applied in delivery systems. The effect of the drying method and starch type is investigated in the next step of this project.

### 3.2. Density and Porosity

**Figure 5:**
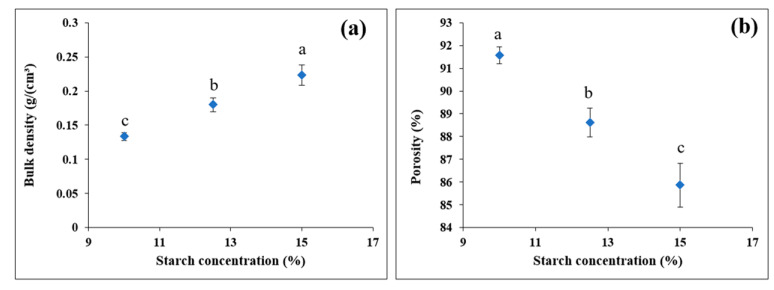
(**a**) Bulk density and (**b**) porosity of 3D-printed starch beads as a function of starch concentration (N = 3).

The density and porosity of the starch beads are represented in Figure 5 as a function of starch concentration. 

No shrinkage was observed during the preparation of starch beads. The high amylose content (72%) resulted in a faster retrogradation and higher gel strength, increasing the resistance to collapse during the drying process. The starch beads revealed low densities ranging between 0.14 and 0.23 g/cm^3^ (Figure 5a), similar to those reported for starch aerogels [7,24]. The porosity of the 3D-printed beads ranged between 86 and 92% (Figure 5b). As expected, the increase in starch concentration resulted in significantly higher density and lower porosity of the beads (*p* < 0.05) (Figure 5); this was also confirmed with SEM images (Figure 3). A similar trend in density and porosity was reported by Zou and Budtova [7] and Buchtová and Budtova [27] for starch cryogels and cellulose aerogels, respectively.

### 3.3. Fourier Transform Infrared Spectroscopy

**Figure 6:**
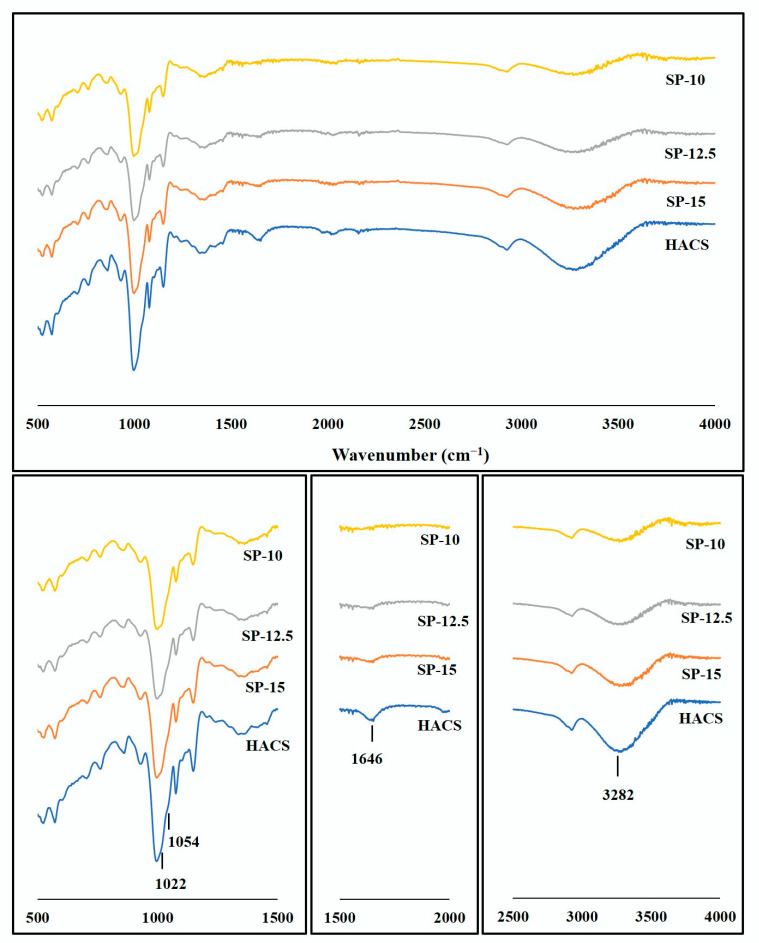
ATR-FTIR spectra of the HACS and 3D-printed starch beads.

The chemical structure of starch beads was elucidated by an FTIR spectroscopy. The FTIR spectra demonstrated identical characteristic peaks; however, their intensities differed slightly (Figure 6).

A strong absorption peak centered at 3282 cm^−1^ was ascribed to the OH stretching mode. The width of this peak implies the hydrogen bonding content. For starch beads, SP-10, SP-12.5, and SP-15, OH stretching peak shifted slightly to a higher wavenumber (3294, 3288, and 3286 cm^−1^, respectively), which can be related to the reduction in crystalline structure [31,32]. The absorption peak that appeared at 2923 cm^−1^ was attributed to the –CH2 stretching vibration. The C–H bending of CH2 revealed IR peaks at 1433 and 1409 cm^−1^. The other bands at 1150, 1105, and 990 cm^−1^ were assigned to C–O, C–C, and C–O–H stretching vibrations, respectively [33]. The characteristic peak that appeared at 864 cm^−1^ was associated with C–O–C at the β-glycosidic linkage [18].

In addition, the starch crystallinity was determined by FTIR spectra by characterizing the changes in the amorphous and crystalline domains. As seen in Figure 6, the strong IR peak at 995 cm^−1^ revealed two shoulders at 1022 cm^−1^ and 1054 cm^−1^, which exhibited the amorphous and crystalline bands, respectively. The higher intensity of the shoulder appeared at 1022 cm^−1^, which suggests the greater extent of short-range double helices [18]. The intensity of this peak increased in the IR spectra of starch beads, confirming the reduced crystallinity. The intensity ratio of these two shoulders as 1054/1022 cm^−1^ was also quantified to compare the crystallinity of the starch beads. This ratio decreased in starch beads compared to HACS, confirming the loss of crystalline structure during gelatinization. For HACS, SP-10, SP-12.5, and SP-15, the 1054/1022 cm^−1^ was calculated as 1.48, 1.23, 1.27, and 1.34, respectively.

The single peak that appeared at1646 cm^−1^ was attributed to the water tightly bound through hydrogen bonding as part of the crystalline structure [33]. This peak almost disappeared in the 3D-printed starch beads, suggesting the reduction in crystallinity after gelatinization.

### 3.4. X-ray Diffraction

**Figure 7:**
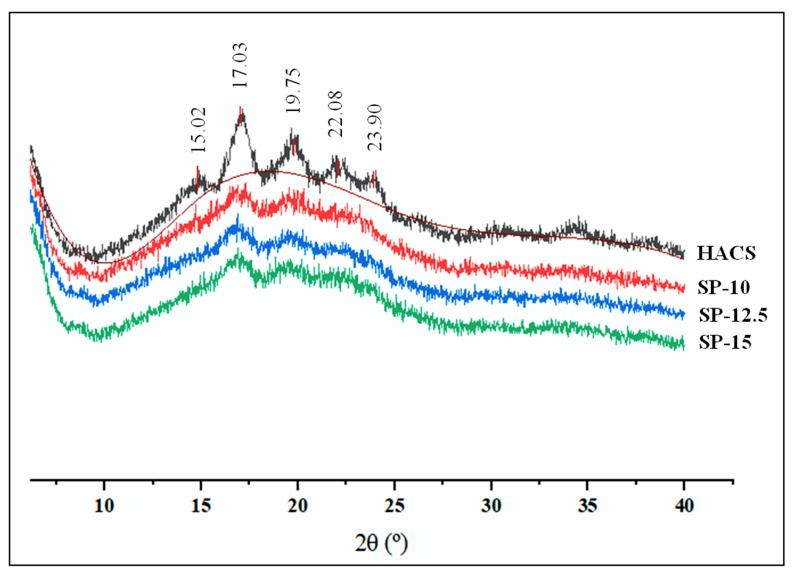
XRD patterns of the HACS and 3D-printed starch beads produced using starch concentrations of 10, 12.5, and 15% (*w*/*w*).

The diffraction profiles of HACS and 3D-printed starch beads are presented in Figure 7. The crystalline part of the HACS was displayed by five diffraction peaks that appeared at 2*θ* = 15.02°, 17.03°, 19.75°, 22.08°, and 23.90°, representing a B-type XRD pattern. The previous studies reported that high amylose starches typically show a B-type XRD pattern, which is identified by five XRD peaks at 2*θ* = 15.26°, 17.21°, 19.75°, 22.32°, and 24.08° [33].

As expected, the intensity of the sharp peaks observed in the XRD pattern of HACS became significantly weaker and broader, indicating the decrease in crystallinity after the gelatinization for all three concentrations [34,35]. This also agrees with the FTIR data reported in Figure 6. The relative crystallinities of HACS, SP-10, SP-12.5, and SP-15 were 8.78, 5.64, 6.33, and 7.33%, respectively. The crystallinity of SP-15 was slightly higher than that of SP-10 and SP-12.5, probably due to the heavier starch recrystallization during retrogradation. These results are consistent with the crystallinity of starch aerogels reported in previous studies [34]. 

### 3.5. Thermal Properties

**Figure 8:**
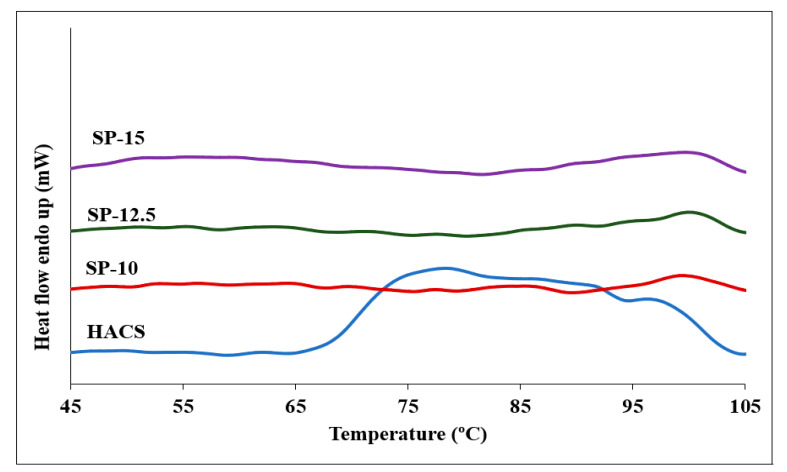
DSC profiles of the HACS and 3D-printed starch beads generated using starch concentrations of 10, 12.5, and 15% (*w*/*w*).

Two endothermic peaks were observed in the DSC thermogram of HACS, with the main peak appearing at 70 °C and the other smaller peak at 95 °C (Figure 8). According to the previous research on starch [36,37], the first endotherm peak appears due to the melting of the amylopectin crystalline structure and/or disrupting the double helices formed in the amylopectin side chains, while the second DSC endothermic peak is associated with the melting of amylose–lipid complexes. Contrary to the melting of amylose, the amylopectin phase transition is irreversible. In a previous study, the peak ascribed to the amylopectin melting disappeared when the starch dispersions were rescanned by DSC, and only the amylose melting peak appeared at 95 °C in the endothermic profile [36,37], which is consistent with our findings.

As depicted in Figure 8, the first peak almost disappeared in DSC thermograms for starch beads confirming the irreversible melting of amylopectin crystalline structure through gelatinization and 3D printing. Our findings agree with other studies reporting the gelatinization temperatures of starches with different amylose contents [38,39]. They reported that high amylose content could cause the unordered amylose chains to agglomerate in the amorphous lamellae and the amylose tie-chains to accumulate in ordered crystalline lamellae, which may weaken the crystalline order by disturbing the double-helical packing. 

Generally, starches have a lipid content between 0.5 and 1.5% [40,41]. Therefore, the lipid content is considered as the other parameter affecting the thermal properties of starches. During starch gelatinization, amylose–lipid complexes are formed since amylose has the innate ability to bind lipids. Amylose–lipid endotherms in HACS and starch beads were observed at 97.3 °C and 101.4 °C, respectively [36,37].

### 3.6. Rheological Properties

The rheological behavior of the bio-ink is critical for extrusion-based 3D printing. The desired properties, including shape stability and resolution, highly depend on the rheological properties of the printing material. Specifically, the ink should show shear-thinning behavior with appropriate flow stress to be printable using an extrusion-based 3D printer, especially with a small nozzle size [42,43,44,45]. Starch pastes consist of entangled amylose molecules creating a continuous network strengthened by swollen granules [16,19]. This specific structure grants starch pastes the required viscoelastic properties (i.e., a shear-thinning behavior) for extrusion-based 3D printing. 

#### 3.6.1. Viscosity

**Figure 9:**
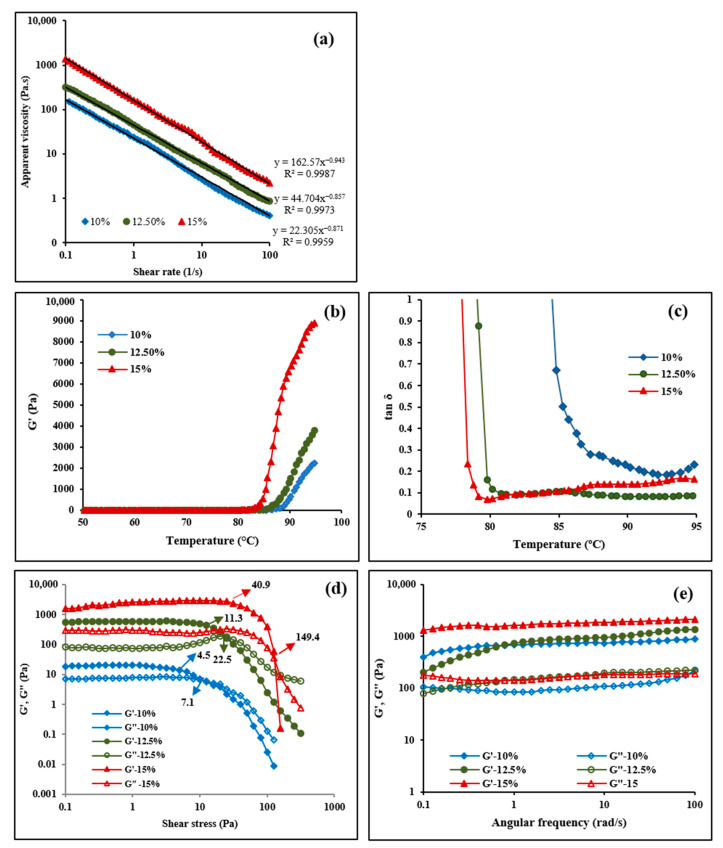
(**a**) Viscosity as a function of shear rate at 95 °C; (**b**) G′ and (**c**) tan δ versus temperature; (**d**) stress; and (**e**) frequency dependence of G′ and G″ for starch suspensions at various concentrations.

The steady shear rheological parameters were measured to investigate the viscoelasticity of starch suspensions as the ink for extrusion-based 3D printing. At a constant shear rate, the viscosity increased as the starch concentration increased (Figure 9a). The double logarithmic plot of various starch concentrations shows a linear correlation between viscosity and shear rate. Therefore, the rheological behavior was described using the power-law model [16,19,46,47,48].

η = Kγ^n−1^ (1)

where η, K, γ, and n are the viscosity (Pa.s), the consistency (Pa.s), the shear rate (s^−1^), and the power-law index, respectively. The power-law parameters are presented in Table 3. For all three starch concentrations, n is smaller than 1, indicating a non-Newtonian fluid that shows shear-thinning behavior. At the same shear rates, the viscosity increased by increasing the starch concentration.

#### 3.6.2. Temperature Sweeps

Storage modulus (G′) and tan δ are two parameters reflecting the viscoelastic characteristics of starch paste. The gelatinized starch obtained by increasing the temperature of a starch solution was able to respond to the elastic deformation and support the printed object [16,19]. The relationship of G′ and tan δ with temperature for three starch concentrations are shown in Figure 9b,c, and Table 3. A similar profile was observed for all specimens during the temperature sweeps. Since the starch remained undissolved in cold water, G′ did not change at low temperatures. By increasing the temperature beyond a certain point (80–90 °C), G′ extensively increased, and tan δ dramatically decreased due to the swelling of granules resulting in the formation of a closely packed network [16,49]. Heating elevated the swelling of granules and amylose leaching out, which led to the formation of a 3D matrix through the collision of amylose chains and swollen starch granules, which contributed to the increase in G′ values.

Higher starch concentrations resulted in higher G′ values (Figure 9b, Table 3). Based on the previous studies, G′ values higher than 500 Pa and tan δ smaller than 0.2 describe starch suspension as an elastic gel [50]. The gel with 15% starch concentration was stiffer than the ones obtained with lower starch concentrations (10 and 12.5%); therefore, it provided better control over shape fidelity, making it more desirable as a bio-ink for 3D printing.

#### 3.6.3. Stress Sweeps

The highest starch concentration (15%) revealed the highest G′ (Figure 9d). Starch hydrogels with 10, 12.5, and 15% concentrations yielded stress values of 4.5, 11.3, and 40.9 Pa, respectively. As a critical parameter to support the shape of the printed object, the mechanical strength of the printing material is reflected by the yield stress [51]. The yield stress is affected by the swelled granules and the amylose chains, as discussed for G′. The point at which G′ and G″ values are equal was also determined as the flow stress presenting the extrudability of the ink based on the force required for extrusion. As seen in Figure 9d and Table 3, yield stress and flow stress were concentration-dependent, agreeing with the previous studies [16]. The 15% starch hydrogel had higher yield stress indicating higher printability and resolution due to the stronger mechanical characteristics, while its higher flow stress required a stronger force (i.e., higher printing pressure) for printing through the nozzle compared to the lower concentrations. The higher yield stress values led to the higher resistance of the starch hydrogels to deformation and produced 3D-printed beads with a higher resolution and no defects.

#### 3.6.4. Frequency Sweeps

The frequency sweeps of the storage (G′) and loss (G″) moduli at 95 °C are depicted in Figure 9e. The mechanical strength of the starch hydrogels is denoted by G′, while the viscous response of the hydrogels is measured by G″. For all samples, G″ was smaller than G′, indicating the dominant elastic properties of the system. At a constant angular frequency, G′ and G″ increased as the starch concentration increased, indicating that the viscoelastic properties of the starch hydrogels correlated with the starch concentration. This caused the SP-10 to deviate from the spherical shape because the mechanical strength was not high enough to support the shape of the 3D-printed beads. Overall, the starch concentration of 15% was the best concentration among the samples investigated to produce small starch beads using 3D food printing.

## 4. Conclusions

In the present study, extrusion-based 3D food printing was used to fabricate spherical porous starch beads. Three-dimensional food printing parameters, namely starch concentration (10, 12.5, and 15%, *w*/*w*) and nozzle size (0.33, 0.25, 0.15, 0.10, and 0.08 mm) were investigated and optimized for the smallest bead size. The smallest bead size (~650 µm) was achieved using a 15% starch concentration with a nozzle size of 0.08 mm. Moreover, the sphericity of the beads increased with increasing the starch concentration; as starch pastes’ viscosity and storage modulus increased with starch concentration, resulting in a better shape fidelity (i.e., spherical shape) at 15% starch concentration. However, higher starch concentration led to higher density (0.23 g/cm^3^) and lower porosity (86%). All the 3D-printed starch beads showed an open porous structure. However, a nonporous layer covering the 3D-printed beads was observed, and its thickness increased as the starch concentration increased, which may provide extra protection in encapsulation applications. The crystallinity of starch decreased mainly due to the gelatinization process involved prior to and during 3D printing. DSC thermograms indicated that the amylopectin crystalline structure was irreversibly melted due to gelatinization. Overall, the proposed 3D food printing approach provides precise control over the shape, size, and composition of the 3D-printed beads while eliminating the need for surfactants, organic solvents, and extra extraction steps to generate starch beads. The porous structure of the 3D-printed starch beads enables them to be used for the delivery of bioactive compounds/drugs in the food and pharmaceutical industries.

## Figures and Tables

**Table 1:** The 3D food printing parameters.

Sample	Starch Concentration (%, *w*/*w*)	Extrusion Temperature (°C)	Nozzle Size (ID, mm)
A1	10	95	23G (0.33)
A2	10	95	25G (0.25)
A3	10	95	30G (0.15)
A4	10	95	32G (0.10)
A5	10	95	34G (0.08)
B1	12.5	95	23G (0.33)
B2	12.5	95	25G (0.25)
B3	12.5	95	30G (0.15)
B4	12.5	95	32G (0.10)
B5	12.5	95	34G (0.08)
C1	15	95	23G (0.33)
C2	15	95	25G (0.25)
C3	15	95	30G (0.15)
C4	15	95	32G (0.10)
C5	15	95	34G (0.08)

**Table 2:** The mean (a) bead size and (b) sphericity of the 3D-printed starch beads.

Sample	Nozzle Diameter				
	23G	25G	30G	32G	34G
	(a) Mean bead size (µm) *				
**SP-10**	3501 ± 440 ^a,A^	3061 ± 502 ^b,A^	1814 ± 207 ^c,A^	1203 ± 319 ^d,A^	812 ± 91 ^e,A^
**SP-12.5**	1869 ± 101 ^a,B^	1272 ± 103 ^b,B^	1151 ± 85 ^c,B^	870 ± 89 ^d,B^	712 ± 82 ^e,B^
**SP-15**	1507 ± 75 ^a,B^	1158 ± 93 ^b,B^	1006 ± 76 ^c,C^	842 ± 103 ^d,B^	650 ± 55 ^e,B^
	(b) Sphericity *				
**SP-10**	0.74 ± 0.04 ^a,A^	0.72 ± 0.05 ^a,A^	0.70 ± 0.06 ^a,A^	0.71 ± 0.03 ^a,A^	0.70 ± 0.03 ^a,A^
**SP-12.5**	0.88 ± 0.01 ^a,B^	0.80 ± 0.03 ^b,B^	0.78 ± 0.04 ^b,B^	0.79 ± 0.03 ^b,B^	0.77 ± 0.03 ^b,B^
**SP-15**	0.98 ± 0.01 ^a,C^	0.86 ± 0.02 ^b,C^	0.87 ± 0.02 ^b,C^	0.90 ± 0.05 ^b,C^	0.87 ± 0.02 ^b,C^

* Means with different lowercase letters within the same column and means with different capital letters within the same row are significantly different according to the LSD test (*p* < 0.05). Fifty measurements were conducted for each replication (N = 3). The sphericity is equal to 1 for spheres, whereas it is <1 for other shapes differing from the spherical shape [20]. However, since the measurement of sphericity requires a careful determination of volumes and surface areas of the beads, commonly, the circularity as a two-dimensional analog is assessed instead. The sphericity depends on the shape and aspect ratio of the beads and can be different from the circularity. However, as reported in the literature [20], these two shape factors can be reasonably approximated as equal for spheroid-like beads. Therefore, in this study, the starch beads’ sphericity was evaluated through the measurement of circularity using ImageJ software.

**Table 3:** Gel strength, loss tangent, yield stress, flow stress, and power-law parameters of the starch pastes with different concentrations.

Sample	Rheological Parameters *				Power-Law Parameters *		
	G′ (Pa, at 95 °C)	Tan δ (at 95 °C)	Yield Stress (Pa)	Flow Stress (Pa)	n	K	R^2^
**SP-10**	2216 ± 115 ^a^	0.215 ± 0.0169 ^c^	4.5 ± 0.5 ^a^	7.12 ± 0.81 ^a^	0.129 ^b^	22.3 ^a^	0.996
**SP-12.5**	3851 ± 528 ^b^	0.088 ± 0.004 ^a^	11.3 ± 1.2 ^a^	22.53 ± 2.58 ^a^	0.143 ^c^	44.70 ^a^	0.996
**SP-15**	8947 ± 292 ^c^	0.160 ± 0.004 ^b^	40.9 ± 8.8 ^b^	142.2 ± 16.3 ^b^	0.057 ^a^	162.57 ^b^	0.997

* Means with different lowercase letters within the same column are significantly different according to the LSD test (*p* < 0.05) (N = 3).

## Data Availability

The data presented in this study are available in the article.
